# Nicotinic augmentation of anti-inflammatory GSK3b signaling

**DOI:** 10.1186/1617-9625-12-S1-A15

**Published:** 2014-06-06

**Authors:** David A Scott, Richard J Lamont, Akhilesh Kumar, Huizhi Wang

**Affiliations:** 1 School of Dentistry, University of Louisville, Louisville, Kentucky, 40292, USA

## Background

Glycogen synthesis kinase 3β (GSK3β) has been shown to be a critical mediator of the intensity and direction of the innate immune system responding to bacterial stimuli. Stimulation of the anti-cholinergic anti-inflammatory system by tobacco alkaloids (nicotine; cotinine) leads to phosphorylation and inactivation of GSK3β and, subsequently, to immune suppression. This presentation will review the tobacco-induced dysregulation of GSK3β signaling and provide insight into the increased susceptibility of smokers to multiple bacterial diseases, including those caused by Mycobacterium tuberculosis, Legionella pneumophila, and Neisseria meningitidis. The extensive ongoing efforts to exploit GSK3β for its therapeutic potential in the control of infectious diseases will also be reviewed.

